# An Unusual Presentation of a Massive Pulmonary Embolism with Misleading Investigation Results Treated with Tenecteplase

**DOI:** 10.1155/2015/868519

**Published:** 2015-02-19

**Authors:** David Migneault, Zachary Levine, François de Champlain

**Affiliations:** ^1^Department of Emergency Medicine, Vancouver General Hospital, University of British Columbia, Room 3300 910, West 10th Avenue, Vancouver, BC, Canada V5Z 1M9; ^2^Emergency Department, Montreal General Hospital, McGill University Health Centre, 1650 Cedar Avenue, Room B2.117, Montreal, QC, Canada H3G 1A4

## Abstract

*Background*. There is no foolproof strategy to identify a pulmonary embolism (PE) in the emergency department, and atypical presentations are common. Negative test results may mislead physicians away from the diagnosis of PE. *Objectives*. The current report aims to raise awareness of an unusual presentation of massive PE and its diagnosis and management, in the face of limited evidence in the scientific literature. *Case Reports*. We report the case of a patient with a negative D-Dimer and a negative Computed Tomography contrast angiography of the chest who was diagnosed twenty-seven hours later with a massive PE, as suggested by a bedside echocardiography. The patient was successfully treated with tenecteplase (TNK). *Conclusions/Summary*. Pulmonary embolism frequently presents atypically and is often a diagnostic challenge. There is limited literature about the treatment of massive PE. Further research on bedside echocardiography for diagnosing PE in unstable patients is warranted. In addition, further study into new thrombolytic agents like tenecteplase in the context of massive and submassive PE is warranted.

## 1. Introduction

There is no foolproof strategy to identify cases of pulmonary embolism (PE) in the emergency department (ED). One in every 500 to 1000 ED patients who present to the ED has a PE, and atypical presentations are common [1]. No matter how aggressively one pursues the diagnosis and work-up, it is believed that about 1-2% of patients with PE will be missed. We report the case of a patient with a negative D-Dimer and a negative Computed Tomography contrast angiography (CTA) of the chest who was diagnosed with a massive PE as suggested by a bedside echocardiography and who was subsequently treated successfully with tenecteplase (TNK).

## 2. Case Report

### 2.1. Day 1

A 63-year-old woman known for erythema nodosum, cholelithiasis, hypertension, and remote breast reduction surgery presented to the ED with a two-day history of constant right upper quadrant (RUQ) “tearing/sharp” pain radiating to the back which was worsened with breathing and movement. The pain was accompanied by occasional nausea and anorexia which had begun two weeks earlier. The patient had never experienced similar pain before and denied associated vomiting, chest pain, dyspnea, or fever.

On examination, her vital signs were as follows: temperature 36.5°C, heart rate (HR) 100 beats/min, respiratory rate (RR) 20 breaths/min, oxygen saturation (O2Sat) of 97% on room air, and blood pressure (BP) 145/100 mmHg. The cardiopulmonary examination was normal. The abdominal examination was remarkable only for mild RUQ tenderness without guarding or rebound and negative Murphy's sign. Normal bowel sounds were present and no costovertebral angle tenderness was noted.

Laboratory work-up (CBC, electrolytes, lipase, amylase, LFTs, troponin, and D-Dimer (The assay used for the D-Dimer at our institution is the STA-Liatest D-DI and a result below 0.5 *μ*g/ml is considered normal or negative.)) was all normal except for a total hyperbilirubinemia of 45 *μ*mol/L. The ECG showed few premature atrial contractions with no other significant abnormalities. In order to rule out the diagnosis of acute cholecystitis versus referred pain from T-spine compression fracture, an abdominal ultrasound, chest X-ray (CXR), and thoracic spine X-ray were requested as well as a general surgery consult. The CXR showed blunting of the left costophrenic angle and bibasilar atelectatic changes. The spine X-ray showed only degenerative changes. Analgesia with IV morphine was provided.

The patient was reevaluated ninety minutes later and found to have increased right sided posterior thoracic pain with deep breathing and worsening abdominal pain. The vital signs and the rest of the physical examination remained unchanged. The diagnosis of PE was entertained and a spiral CT scan of the chest with IV contrast using a PE protocol and an infused CT of the abdomen were requested. The CTA of the chest read by the radiology attending staff showed no evidence of clots in the central, segmental, or proximal subsegmental pulmonary arteries. The study was slightly limited by the fact that the left lower lobe segmental pulmonary arteries were not well visualized. Both lower lobes and the lingula were remarkable for subsegmental atelectasis. The CT abdomen was unremarkable overall, showing approximately 15 subcentimetric gallbladder stones with no complication or biliary tree dilatation seen.

### 2.2. Day 2

The next morning, the patient was reassessed and her condition judged to be stable. The abdominal ultrasound was performed and showed the same findings as the abdominal CT, that is, gallstones with no sign of an acute process. Repeat laboratory work-up (CBC, electrolytes, and liver profile) showed only an elevated total bilirubin (45 *μ*mol/L) and an alanine aminotransferase (ALT) of 34. Internal medicine was consulted and they suggested the possibility of early herpes zoster versus background chronic abdominal pain which could be investigated as an outpatient. They specified that there was no evidence of PE, vascular, soft tissue, or rib/bone abnormalities.

### 2.3. Day 3

At 1:35 a.m. the next morning, the patient was found to be confused, moaning, and trembling, with cold extremities. She denied chest pain. Her vital signs were as follows: RR 28/min, O2Sat 88% on room air, BP 92/70 mmHg, HR 141/min, and temperature 36.5°C. The O2Sat increased to 90% on a nonrebreather mask. A venous blood gas was obtained and a femoral line was established. An ECG was done and showed sinus tachycardia with no ST-T changes. The oxygen saturation decreased progressively and the patient developed cyanosis and pallor.

**Figure 1:**
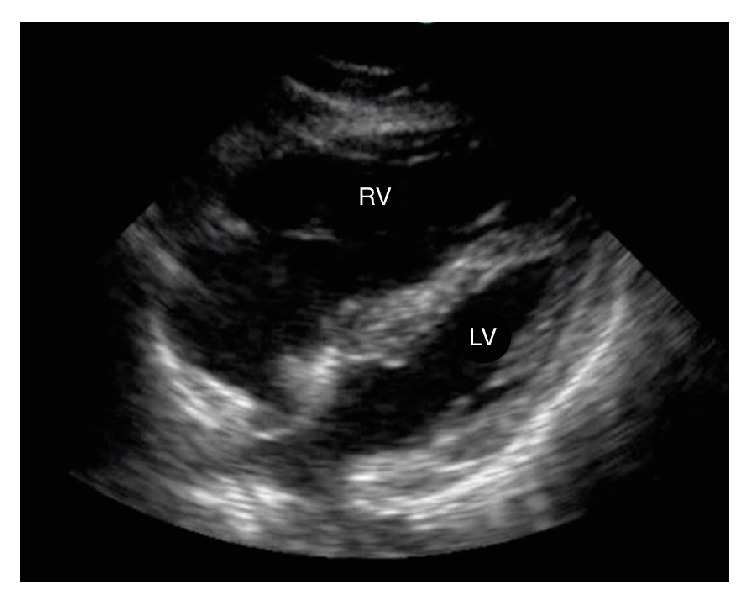
Cardiac ultrasound, subxiphoid view showing a dilated right ventricle (RV).

The decision was made to intubate the patient, the intensive care unit (ICU) was consulted, and some blood work, including a repeat D-Dimer, was ordered. A bedside cardiac ultrasound was performed by an attending emergency physician. It demonstrated a dilated, hypokinetic right ventricle as well as increased right sided pressures as demonstrated by inferior vena cava (IVC) distention greater than 20 mm with no inspiratory collapse (Figure 1).

**Figure 2:**
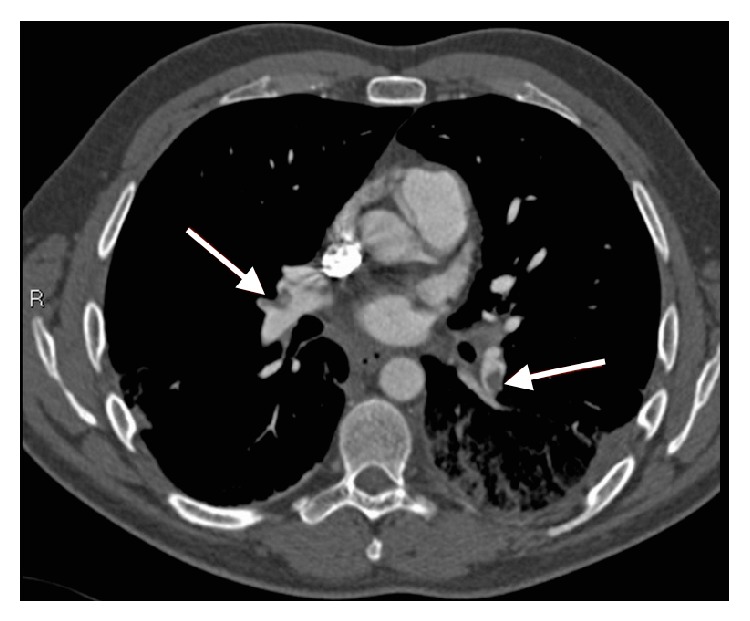
CT pulmonary angiography (CTPA) showinga bilateral pulmonary embolisms with clots in branch arteries.

The D-Dimer came back positive at >4.0 *μ*g/mL. A massive PE was suspected and since it was not possible to obtain a transesophageal echocardiogram (TEE) as a confirmatory test at that time of the night and the patient was too unstable for a repeat CTA of the chest, a decision was made to attempt thrombolysis using tenecteplase (TNK). A dose of 35 mg (7000 U) (0.5 mg/kg (100 U/kg)) was used in conjunction with 5000 U of unfractionated heparin. The patient was then transferred to the ICU. A repeat CTA of the chest was performed and showed bilateral extensive pulmonary embolus (Figure 2).

## 3. Discussion

This case highlights several controversies related to the diagnosis and treatment of a PE in the ED. Herein, we review the diagnostic accuracy of the D-Dimer and CTA of the chest, the use of bedside echocardiography, and the off-label use of tenecteplase for the treatment of PE.

### 3.1. Diagnostic Modalities for PE

Our patient was initially evaluated as low risk for PE. Based on Wells et al., her pretest probability for PE was approximately 3.4% [2]. The literature shows a great variability in the sensitivity and specificity of the D-Dimer. Brown et al. report 93% and 74%, respectively [3]. With a negative D-Dimer, we can infer a posttest probability of approximately 0.33%. The initial decision not to order a CTA was therefore justified. As mentioned earlier, the imaging was later ordered and found to be negative for PE. Again, there is variability in the literature on the sensitivity and specificity of the CTA of the chest. As per PIOPED II, they are 83% and 96%, respectively [4]. Based on those values, we can calculate a reassuring combined negative D-Dimer and CTA of the chest posttest probability of 0.06% for low risk patients.

Another approach which has been proven to be safe and effective is to combine the CTA of the chest with bilateral lower extremity Doppler ultrasonography (US) for all patients with moderate to high pretest probability of PE [5]. Notably, the lower extremity US was not done on this patient in the ED. Hence, it is impossible to know if a positive result would have been found on the initial presentation, prompting earlier therapy. Our case report also raises the question of the relative benefit of adding CT venography to the chest CTA protocol as it is done routinely in some institutions. This combination yields an absolute increase in detection of venothromboembolism (VTE) of 2-3%. This is similar to the additional yield of lower extremity US with CTA chest but at the expense of increased radiation exposure [4, 6–8].

In our case, the finding that triggered treatment was the evidence of a dilated, hypokinetic right ventricle and the increased right sided pressures as demonstrated by the inferior vena cava (IVC) distention on bedside echocardiography. It is important to note that this bedside echocardiogram was performed while the patient was intubated where evaluation of the IVC distention is less specific for the evaluation of elevated right sided pressures. The literature is very limited on the use of a bedside echocardiogram performed by an emergency physician in the diagnosis of PE. Its utilization seems most useful in the situation in which a massive PE is suspected in the periarrest situation, especially when a bedside TEE is not available in a timely manner. In such a situation, the bedside ultrasound can help the physician to narrow the differential diagnosis and to consider further investigations and/or immediate definitive treatment [9].

### 3.2. Thrombolysis for PE

Indications for thrombolysis for PE are controversial and are poorly defined. The risk-benefit analysis suggests that it is of greatest value in the subset of patients with proven PE who are likely to die or to develop circulatory shock or recurrence [10]. In the absence of an evidence based definition, it is generally accepted that a massive PE is defined by a systolic blood pressure less than 90 mm Hg for more than 15 minutes. In the absence of contraindications, patients with proven massive PE probably benefit from thrombolysis. When to administer a fibrinolytic agent to a patient with profound shock remains controversial when the decision is based solely on clinical suspicion of PE derived from information obtained at the bedside (i.e., empirical fibrinolysis in absence of pulmonary vascular imaging).

Administration of alteplase to patients with PE results in more rapid symptomatic improvement than standard antithrombotic therapy alone and causes more rapid normalization of right ventricular function [11, 12]. Tenecteplase is a recombinant plasminogen-activating enzyme that differs from alteplase by its longer half-life, resistance to plasminogen activator inhibitor-1, and increased fibrin specificity, which results in less fibrinogenolysis and less coagulopathy [12]. Its efficacy and safety have not yet been verified by a well-designed randomized clinical trial. However, a literature review reveals 16 case reports where tenecteplase was used as “off-label” to treat PE. Since no standard dose has been determined, the perceived consensus is to use the recommended dose for thrombolytic therapy for acute myocardial infarction [13–28].

A recently published study examining the indications for fibrinolysis in the treatment of submassive pulmonary embolism evaluated the efficacy and safety of single IV bolus of tenecteplase in addition to heparin as compared to heparin alone for normotensive patients with acute PE who have echocardiographic and laboratory evidence of right ventricular dysfunction. Hemodynamically unstable patients were being excluded. Nevertheless, they concluded that fibrinolytic therapy prevented hemodynamic decompensation but increased the risk of major hemorrhage and stroke. A more recent meta-analysis similarly concluded that thrombolytic therapy was associated with lower rates of all-cause mortality and increased risks of major bleeding and ICH. These results are likely not applicable to our unstable patient [29, 30].

## 4. Conclusion

Pulmonary embolism in the context of a negative D-Dimer and a negative CT chest, although uncommon, does occur, and emergency physicians should be aware of this possibility. Bedside echocardiography can help the emergency physician support the diagnosis of PE in hemodynamically unstable patients. Tenecteplase is a treatment option to consider in such patients with suspected PE, as reported in this case and in other published case reports. Further research on the diagnosis and treatment of massive and submassive PE is clearly warranted.
